# Advances in Molecular Tools and In Vivo Models for the Study of Human Fungal Pathogenesis

**DOI:** 10.3390/microorganisms8060803

**Published:** 2020-05-26

**Authors:** Dhara Malavia, Neil A. R. Gow, Jane Usher

**Affiliations:** Medical Research Council Centre for Medical Mycology, University of Exeter, Geoffrey Pope Building, Stocker Road, Exeter EX4 4QD, UK; d.malavia@exeter.ac.uk (D.M.); n.gow@exeter.ac.uk (N.A.R.G.)

**Keywords:** fungal pathogens, CRISPR, RNAi, genetic tools, animal models

## Abstract

Pathogenic fungi represent an increasing infectious disease threat to humans, especially with an increasing challenge of antifungal drug resistance. Over the decades, numerous tools have been developed to expedite the study of pathogenicity, initiation of disease, drug resistance and host-pathogen interactions. In this review, we highlight advances that have been made in the use of molecular tools using CRISPR technologies, RNA interference and transposon targeted mutagenesis. We also discuss the use of animal models in modelling disease of human fungal pathogens, focusing on zebrafish, the silkworm, *Galleria mellonella* and the murine model.

## 1. Introduction

Fungal pathogens have long been associated with infectious diseases in humans and other mammals [1,2,3,4]. At least one-quarter of the human population will experience a superficial mucosal fungal infection during their lifetime, however, life-threatening infections occur less frequently but now at an increasing rate due to medical advancements and ability of populations to have longer lifespans and patients to survive trauma and immunotherapies [1,5]. To date, no vaccines have been approved for human use to halt the rise of fungal infections and so our ability to understand fungal disease and to develop novel antifungal interventions is vital. Over the past decades, the burden of human fungal disease has increased, and researchers have developed a plethora of techniques to investigate fungal pathogenesis, host immune responses, antifungal drug development, the genetics of pathogens. Here we focus on three of these tools, CRISPR-Cas gene editing, RNAi and transposon mutagenesis; that are rapidly improving our understanding of the lifestyle of pathogenic fungi. We also discuss the use of a small repertoire of different animal models, that have been utilized to test emerging hypotheses from these studies in terms of the analyses of potential drug targets, novel inhibitors and immunotherapies. In Table 1, we have highlighted the various tools and resources that will be addressed in this review and animal models of which the most popular we shall discuss further.

## 2. CRISPR/Cas Gene Editing

**Figure 1:**
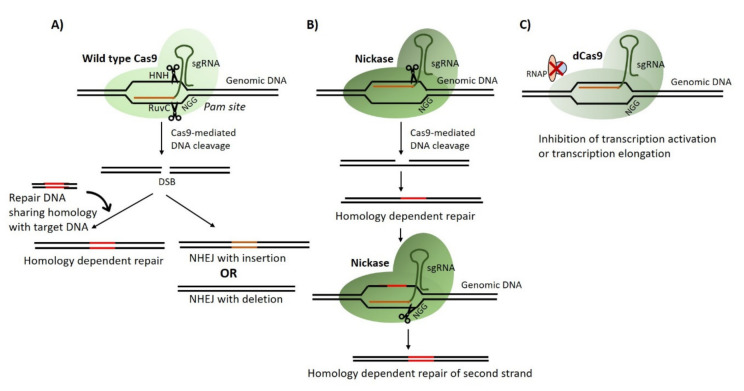
Schematic representation of CRISPR-Cas mediated gene editing. (**A**) Cas9 forms a complex with sgRNA and binds to the target DNA. Active sites on Cas9 (HNH and RuvC) create a double-stranded break (DBS), which is repaired by homologous recombination or non-homologous end-joining (NHEJ). Variants of Cas9 include: (**B**) Nickase which lacks one of the active sites (HNH or RuvC) resulting in cleavage of one target strand at a time and; (**C**) dCas9 or dead Cas9 which prevents transcription by inhibition of either initiation or elongation of the target transcript. (Figure adapted from Tian 2017 Synthetic and systems biology; Doudna and Charpentier 2014 Science; Satomura 2017 Scientific reports).

The discovery and application of CRISPR technology has been a valuable addition to the tools for gene editing in pathogenic fungi particularly in diploid fungal species that lack meiosis and transfectable plasmids. Briefly, gene editing is achieved by the introduction of three components into the cell that is to be edited: (i) a bacterial nuclease (commonly Cas9 or Cas12a); (ii) a guide RNA for targeting of the nuclease; (iii) an exogenous donor DNA containing the desired mutation and region of homology with the target site. The guide RNA transports the nuclease to a specific target site on the genome, where the nuclease generates a double-stranded break (DSB). Cleaved DNA is then repaired either via non-homologous end joining (NHEJ) or in the presence of a donor (repair) DNA via homology-directed repair (HDR) (Figure 1) [6,7,8,9,10]. Subsequently, a number of studies have employed CRISPR/Cas9 strategy to study genes that may contribute to fungal virulence [11,12,13,14,15,16,17,18,19]. Interestingly, Kildergaard et al., combined the CRISPR/Cas9 system with RNA-interference (RNA-i) to generate simultaneous mutations in multiple genes [20]. Similarly, CRISPR/Cas9 systems have been employed to generate multiple simultaneous mutations with high efficiencies [21,22,23,24]. In addition, CRISPR technology has also been adopted for genome-scale engineering to produce and test the phenotypes of genome-wide mutations in parallel. Workflows such as CHAnGE [25], MAGESTIC [26] and CREATE [27] have been developed in *Saccharomyces cerevisiae* for precision large scale gene editing. These expression cassettes also incorporate unique barcode sequences for tracking mutants in relative fitness experiments.

CRISPR technology is not limited to gene editing via DSBs alone. Recent studies have demonstrated that nuclease dead variant of Cas9 known as dCas9 can be employed as a means of over-expressing or repressing transcriptional activity and introducing base substitution mutations [28,29,30]. Another modification in Cas9 involves inactivation of one of the two active domains (HNH or RuvC) of the nuclease enzyme thus allowing single-stranded breaks to be introduced at the target site with consequential minimizing off-target effects [31,32]. While most of these Cas9 variants have demonstrated high on-target mutation efficiencies in *S. cerevisiae*, their application can readily be extended to other fungal pathogens. Recently, Roman et al. employed dCas9 fused with transcriptional activator Gal4 and/or VP64 and repressor Nrg1 to regulate expression of catalase gene *CAT1* in *Candida albicans* [33]. Wensing and colleagues also developed an improved dCas9 system fused with codon optimised repressor domain of mammalian repressor Mxi1. The authors established an efficient and scalable CRISPR-interference (CRISPRi) system for *C. albicans* which when paired with corresponding guide RNA, can allow repression of the desired gene [34].

Gene drives are systems of inheritance that enable the propagation of the desired set of genes throughout a population. Gene drives typically deploy the introduction of CRISPR RNA and Cas9 to increase the probability that a specific allele will be transmitted to the offspring or progeny. Recently, CRISPR technology has been used to develop gene drive systems in fungi amongst other organisms such as mice and mosquitoes [35,36,37,38]. A fundamental design of CRISPR based gene drive employs a nuclease (Cas9 or Cas12a), guide expression cassette(s) integrated into the genome and donor (“cargo”) element. The CRISPR/Cas construct is copied via DSB and homology-directed repair allowing the integration of the “cargo” element and converting cellular heterozygosity to homozygosity [39]. Such gene drives follow super-Mendelian inheritance (above 50%) allowing biased propagation of desired genetic elements within a population and eventually the entire species [38,40]. Recent studies have shown the efficiency and success of CRISPR gene drives expanding their potential application in studying fungal pathogens [41,42,43]. Recently, Shapiro and colleagues used CRISPR/Cas9 gene drive system to generate double-gene deletion libraries in *C. albicans* targeting drug efflux pumps and adhesion factors [44]. Subsequently, Halder and colleagues developed an optimized protocol for CRISPR based gene-drive system to study genetic interactions in *C. albicans* [45]. Furthermore, improvements in CRISPR-Cas9 gene drives have been developed providing means to markerless-selection, bias towards homology-directed repair, increased layers of biosafety, maximizing potential redundancy and regulating gene-drive activity [39,41,42,46].

Traditional CRISPR/Cas9 technology has drawbacks, which include inefficient sgRNA design, undesirable repair by NHEJ and off-target mutagenesis associated with the ability of Cas9 to tolerate some mismatch between sgRNA and target site [47,48]. However, with an enhanced understanding of CRISPR/Cas technology, improvements have been made that ensure high on-target efficiency and reduction of off-target effects. For instance, flanking sgRNA with 5′ tRNA and expression under a strong RNA polymerase II promoter, ADH1, can improve mutation efficiency by increasing sgRNA expression [49,50]. Recently, Cas12a (also known as Cpf1) was introduced as a replacement for Cas9 nuclease increasing the repertoire of CRISPR gene editing toolbox for *Saccharomyces* [51]. Furthermore, the mutational substitution of the C-terminal domain or deletion of *DNL*4/*LIG*4 results in partial or complete loss of NHEJ resulting in HDR bias following cleavage by Cas9 [14]. Other advancements in the field include having the guide RNA expression cassette housing unique restriction enzymes sites [52], markerless selection [53,54], a single plasmid carrying guide and repair DNA (e.g., unified plasmid) [55], PCR-free mutagenesis [56], cloning-free in vivo assembly [57,58,59] and rapid cloning in a single reaction [60].

More recently, prime editing was introduced in mammalian systems, which involved a modified dCas9 fused with engineered reverse transcriptase and corresponding prime editing guide RNA (pegRNA) that simultaneously encodes for the desired mutation and ensures precise DNA targeting. Prime editing offers gene editing without creating DSBs or donor DNA [61]. Additionally, Cas13 has been developed as an RNA editing nuclease, which allows RNA knockdown and precise editing in mammalian cells [62]. While these more recent advancements have been demonstrated in mammalian cells, they can potentially be exploited to study fungal biology and pathogenesis [63].

## 3. RNAi

**Figure 2:**
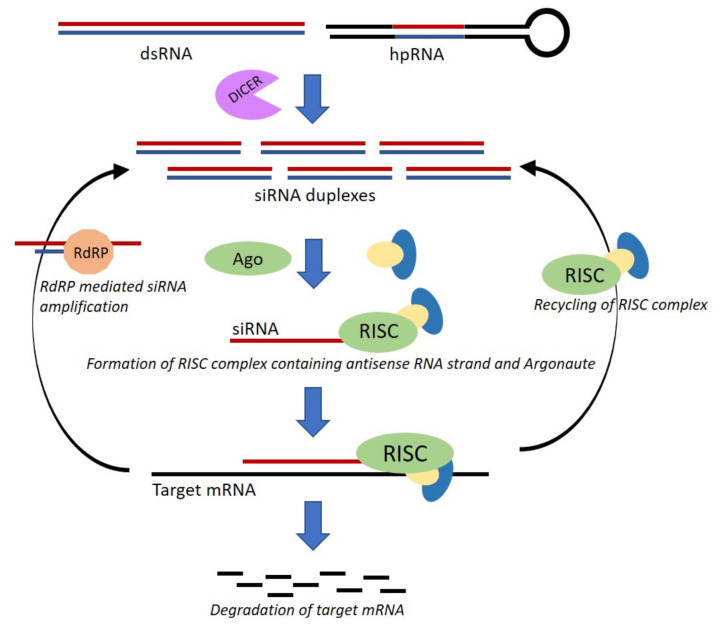
Schematic representation of RNAi mediated silencing in fungi. Exogenous dsRNA or hpRNA is processed by Dicer into siRNA (~21 nt). The guide siRNA strand binds to Argonaute (Ago) and other proteins to form the RNA-induced silencing complex (RISC). RISC and siRNA complex then binds to target mRNA in homology dependent manner resulting in target degradation. The components of the RISC complex are recycled, and the process is repeated. RNA-dependent RNA polymerase (RdRP) is found in some fungal species, which functions by regenerating siRNA duplexes allowing sustained silencing. (Figure adapted from Majumdar, Cary 2017 and Schumann 2010).

RNA interference is an evolutionarily conserved mechanism found in most eukaryotes including many fungi. It confers protection against exogenous and endogenous genetic threats and regulates gene expression. RNAi is initiated by the introduction of a double-stranded RNA (dsRNA), which is homologous to the target sequence. The dsRNA is processed to 21–25 nucleotides by an endonuclease known as DICER. One strand of the resulting small interfering RNAs (siRNA) associates with the effector protein, Argonaute, which then binds and degrades target mRNA in a homology dependent manner allowing silencing of gene transcript levels (Figure 2) [64,65]. RNA dependent RNA polymerase (RdRP) is an enzyme found in certain fungi that is thought to amplify siRNA to allow sustained target silencing [66]. Other small RNAs that induce silencing include micro-RNA (miRNA), hairpin RNA (hpRNA), trans-acting small interfering RNAs (tasiRNA) and qiRNAs (Qde-2 associated RNAs). These small RNAs originate from dsRNA and use the common RNAi machinery [67]. A number of studies on model fungi like *Neurospora crassa* and *Schizosaccharomyces pombe* have improved our understanding of RNA interference in eukaryotes. In this section, we will focus on an application of RNAi as a molecular tool for genetic research in pathogenic fungi while highlighting the advantages and shortcomings of this technique [68,69,70,71,72].

RNAi has been used as a gene silencing strategy in a number of pathogenic fungi. These include *Cryptococcus*, *Candida*, *Aspergillus*, *Sporothrix*, *Histoplasma capsulatum*, *Blastomyces dermatitidis* and *Debaromyces hansenii* [73,74,75,76,77,78,79,80,81,82,83,84]. Most of these pathogenic organisms possess key components of RNAi, which include Dicer and Argonaute proteins, thus allowing the exploitation of RNAi strategy for fundamental cell biology, metabolic engineering and to study virulence. Additionally, some reports have generated RNAi tools for future studies. For example, Skowyra and Deoring were among the first to establish an RNAi system in *C. neoformans*. They generated two RNAi constructs allowing constitutive and inducible expression of dsRNA and hence, constitutive and inducible silencing of target genes [85]. In another study, a synthetic RNAi system was developed in *C. glabrata* by introducing Argonaute and Dicer genes from *Saccharomyces castelli*. Using this RNAi strain as a springboard, silencing constructs were designed to generate and screen a gene library for previously unidentified genes that play a role in cell integrity, ROS resistance and virulence [86]. In more recent years, RNAi system has been reported in *Paracoccidioides* as well. While initial attempts to apply RNAi technology in *Paracoccidioides* resulted in unstable gene silencing [87], the introduction of intron-containing hairpin construct allowed successful knockdown of the target gene [88]. Despite the successful application of RNAi in the aforementioned fungi, it has been difficult to establish the system in some others. For example, while a Dicer-like protein, DCL1, has been identified in *Coccidioides immitis,* RNAi has not been established for genetic manipulation. Similarly, RNAi components have been identified and studied in *Mucor circinelloides* but have not been exploited for artificial gene regulation [72,89,90].

Although model yeast *S. cerevisiae* lacks its own RNAi machinery, it has been shown that the introduction of Argonaute and Dicer from an RNAi proficient organism like *S. castelli* allows enforcement of RNAi in *S. cerevisiae* [91,92,93]. The establishment of an artificial RNAi system in *S. cerevisiae* has improved our understanding of this molecular tool. It has been demonstrated that gene copy number plays a crucial role in driving endogenous RNA interference. Transcripts from high copy DNA are therefore a favourable property of minimal RNAi systems [94]. Additionally, it was recently shown that Hsp90 regulates conformational changes in Argonaute upon binding to Dicer products in budding yeast [95]. Like Hsp90, Paf1C, a conserved RNA polymerase associated factor 1 complex, also regulates RNAi in budding yeast by preventing siRNA-effector complex from targeting nascent transcripts. These reports provide evidence of factors that regulate RNA silencing, which may be considered while using RNAi as a genetic tool [96]. Subsequently, various RNAi tools have been developed in *S. cerevisiae* to study metabolic regulation on a genome-wide scale [97,98]. While these studies may not yet have direct application in medical mycology, the techniques and tools developed in these reports can be employed in the study of pathogenic fungi, associated virulence factors and host-pathogen interactions.

One of the biggest advantages of RNAi is that it suppresses gene expression in a sequence-specific manner since target recognition is based on base pairing with siRNA. Hence, it can also be used for efficient suppression of alternately spliced gene variants [99]. Additionally, using RNAi strategy it is possible to silence multiple genes simultaneously by targeting a conserved region in a gene family or designing a chimeric construct derived from genes of interest. Gene suppression by RNAi can be variable among simultaneously transformed fungal cells. Hence, by employing a series of RNAi mutants that exhibit different levels of gene suppression, it is possible to study corresponding phenotypes and assess the extent of gene suppression required to generate a desired phenotypic defect [78,100]. Such tools can be effective in the determination of antifungal drug targets. Therefore, RNAi serves as an alternate tool in fungal species where gene manipulation via homology dependent DNA repair and modification is difficult. In addition to these advantages, RNAi-mediated gene manipulation does not alter the structure of the target gene providing a good alternative to traditional gene knockout techniques, especially when knockout of genes essential for survival is difficult [101]. Due to the minimal nature of the RNAi system, it is possible to establish this molecular tool even in fungi that have lost RNAi. This has previously been achieved by the introduction of Dicer and Argonaute genes from RNAi proficient organisms like *S. castelli* or humans [86,95,102,103].

Despite its advantages, a number of drawbacks have prevented the wide-scale application of this technique. One of the major disadvantages of RNAi is the incomplete or reversible gene suppression that is achieved, which results in phenotypic variations different from knockout mutants making it difficult to interpret RNAi data. While RNAi-mediated silencing occurs in a sequence-specific manner, off-target effects, as reported in mammalian cells, may be possible but are yet to be studied in fungi [104,105]. Additionally, genetic complementation cannot be used to verify phenotypes generated through RNAi. As a result, it is impossible to predict whether the phenotypic outcome is the result of suppression of the target gene or arises from an off-target effect. RNAi can be achieved by three methods: (i) expression of a hairpin RNA from a vector; (ii) expression of dsRNA resulting from convergent transcription of a transgene; (iii) by direct introduction of siRNA or dsRNA into a fungal cell [73,76,77].These three approaches are not equally efficient in all fungi. The hairpin/stem-loop approach has been shown to be efficient in *C. neoformans*, but it does not trigger RNAi in *C. albicans* [105]. There is little evidence of the successful direct introduction of siRNA or dsRNA in fungi and is suspected to have low target efficiency [78,105]. The ease of designing constructs and induction of RNAi via generation of sense and antisense RNA are suitable for high throughput screening experiments. However, this method has been shown to have lower efficiency compared to the hairpin RNA approach [106], which, while efficient, are more difficult to design [107]. As a result, the benefits and drawbacks of each RNAi strategy influence the efficiency of gene silencing that is achieved and must be considered during experimental design.

## 4. Transposable Elements

Molecular tools discussed in the previous sections enable targeted mutagenesis. Transposable elements (TEs), on the other hand, serve as a potent and tools for random mutations and genetic polymorphisms. TEs occur as DNA sequences in most organisms and these possess the ability to move and change position within the genome. These transposition events can have a range of consequences from gene inactivation of the landing site gene to the generation of multiple gene copies. Although there are many types of TEs, they are broadly classified as retrotransposons and DNA transposons. Both can be highly mutagenic in nature. A number of reviews provide details on the classification and mechanisms of transposition adopted by each type of TEs [108,109]. One of the drawbacks preventing wide-scale application of TEs in gene manipulation is that the mechanisms and scope of how they affect gene and genome function are not always well understood. Additionally, biases in transposition targeting and insertions can result in an uneven distribution of insertions over the fungal genome [110]. For example, transposable element Tf1 preferentially targets promoters of genes induced by environmental stresses. However, over the past few years, some studies have emerged that contribute to our knowledge of TEs and their mechanisms [111,112]. Despite limitations, transposon-mediated mutagenesis has been used successfully to study protein localization, morphological transitions, phenotypic analysis and to determine genes essential for growth and virulence [113,114,115,116,117,118,119,120]. Most of these studies allow for genome-wide screening by generating a transposon insertion library. Such libraries are typically constructed by mutagenesis of the plasmid-based DNA library, which is then cloned into desired yeast strain [121]. One such widely used transposon insertion library was generated using a minimal plasmid hosting a modified bacterial transposon (Tn7-derived), selection markers for *E. coli* and *S. cerevisiae*, a *lacZ* reporter and Cre-lox recombination sites. This system enables gene disruption and epitope tagging and the mutant library generated in this manner can be used in future functional genomic studies [122]. A tagged heterozygous transposon disruption library was constructed in *C. albicans* that aided gene annotation and identification of potential new drug targets [123]. Additionally, transposon mutagenesis has also been used to study large-scale synthetic genetic interactions referred to as complex haploinsufficiency in *C. albicans* [114,124,125].

Transposon mutagenesis is also used in gene tagging thus facilitating systems-level analyses of non-model organisms [126,127,128,129]. In a recent study, a universal collection of Gateway-compatible 4280 TagModules was constructed as an efficient tool to generate tagged mutants in a range of microbial species. A major advantage of this collection is that it is platform- and organism- independent, thus it can adapt to any DNA tagging strategy including transposon mutagenesis [123]. In another study, Smith and colleagues used a transposon disruption-barcoding strategy to generate a collection of barcoded disruption mutants. They further illustrated the application of their library in the identification of 10,000–1,000,000 gene–gene and drug–gene interactions in a single experiment using microarray-based and next-generation sequencing-based platforms [130].

Genetic studies in non-model fungi including various fungal pathogens are limited as the number of techniques available for generation and identification of gene mutations are limited. To overcome this, a number of autonomous and non-autonomous transposable elements have used to develop genetic tools to study fungal pathogens [128,129,131,132]. For example, an engineered *Aspergillus fumigatus* strain containing *Impala160*, a DNA transposon from *Fusarium oxysporum* was employed to generate and screen a library of insertional mutants. Using this approach, the authors identified 20 previously unknown genes essential for the growth of *A. fumigatus* [10]. While the Impala system is a popular choice, transposition frequency is variable among species. The system was later improved with the discovery of enhanced activation of impala transposon upon prolonged exposure to low temperatures. It was applied to identify 96 loci crucial for the viability of *A. fumigatus* [133]. Meilich et al. also developed a two-element transposon tagging system known as Ac/Ds in *C. albicans* to allow in vivo insertion mutagenesis [134].

In conclusion, although many studies employing transposon mutagenesis are performed in *S. cerevisiae* and *C. albicans*, there is a growing body of work using transposons in other pathogenic fungi including *Cryptococcus*, *C. glabrata* and *Histoplasma* [111,135,136,137,138,139]. Since transposon mutagenesis allows genome-scale DNA modification, this strategy serves a powerful tool to generate insertional mutations.

## 5. Animal Models for Studying Human Fungal Pathogens

Animal models of fungal infection are a key tool to test hypotheses about genes that are potentially important in pathogenesis due to human fungal pathogens. Many different types of animal models of fungal infection have been developed although the murine model remains the most frequently used to study pathogenesis, virulence, diagnosis, immunology and the development of novel therapies. The murine model has been used to mimic a range of human disease states and to monitor disease in a quantitative manner. However, a range of other complementary vertebrate and non-vertebrate models have been integrated into experimental infection biology, each of which confers certain advantages limitations. In this section we will highlight the main models’ systems used in the medical mycology field, highlighting their strengths and weaknesses as no single model can be relied on to answer the many questions one asks in experimental studies or mimic all the various diseases seen clinically.

### 5.1. Zebrafish

Zebrafish have been used for nearly 30 years as a model system to study disease. They are genetically tractable and enable investigation of the innate immune system in isolation from adaptive immunity [140]. Zebrafish share significant genomic homology with humans with 80% of human genes associated with orthologues present in the zebrafish. These include counterparts of mammalian pathogen recognition receptors (PRRs) such as Toll-like receptors (TLRs) and nucleotide-binding oligomerisation domain-like receptors (NLRs)—and these have been shown to play important roles in zebrafish host defences [141].

A wealth of zebrafish transgenic lines exist, many of which express fluorescent proteins notably in key cell types such as phagocytes. This has allowed researchers to study dynamic interactions between macrophages, neutrophils and other immune cells with human fungal pathogens such as *Aspergillus, Candida* and *Cryptococcus* species [142,143]. In the *Aspergillus* field, zebrafish have been used to characterise the different host responses against slow and fast germinating strains. More recently, work with zebrafish has focused on the emerging drug-resistant fungal pathogen *Candida auris. (C. auris)* infections do not result in neutropenia in vivo, as NETs are not produced to circumvent the infection [144]. The zebrafish model has also been extensively used to study host defence [140] trained innate immunity [141,145], which is based on the development of ‘memory’ for pathogens after infection via epigenetic reprogramming. Previous studies in the mouse model showed that trained immunity priming with low doses of *C. albicans* can protect against secondary infections in a macrophage dependent and T cell-independent manner [145].

The use of zebrafish in the area of medical mycology has increased over the last decade and has taken advantage of the development of cutting edge real-time microscopy techniques and the ability to obtain fish embryos in large numbers, enabling high-throughput screens to be undertaken [144,146,147,148,149,150]. This has benefited hugely from the status of the zebrafish larva as a model system at the forefront of in vivo cell biology, and the analysis of 4-d cellular dynamics resolved at a single-cell level [150]. Zebrafish will not replace the vertebrate model of mice in immunology, but it can be utilised to investigate fundamental concepts in pathogenesis and host defence and potentially aid in the development of novel therapies to combat human fungal pathogens.

### 5.2. Bombyx mori—The Silkworm

The silkworm has various advantages as an experimental animal, such as the low cost of rearing and fewer ethical issues [151,152,153]. Various strains of silkworm and rearing methods have been established in the long history of sericulture, resulting in large numbers of silkworms being easily reared in a small place. The silkworm is larger than fruit flies and nematodes, it is easy to perform experiments that require injections of fungal burdens [151,154]. In addition, the intra-hemolymph and intra-midgut can be easily distinguished and used as separate sites of inoculation with the former providing an analogous system to an intravenous injection and the later to oral administration in humans. This is in contrast to the *G. mellonella* larvae that are smaller which makes accurate injection into the intra-midgut more challenging [151]. Using the silkworm is advantageous as an experimental animal, as it is possible to study molecular mechanisms of infection by human fungal pathogens, as we will discuss in this section. However, a limitation of all insect models is that they do not survive freezing, therefore, requiring continuous breeding and maintenance to provide a source of materials.

The use of the silkworm has been included in infection studies with *C. albicans*, *C. glabrata, C. neoformans, and A. fumigatus* [155,156,157]. It has been reported that silkworms die when incubated at 27 °C post-infection when *C. albicans* is injected into their hemolymph [155]. Additionally, the administration of antifungal drugs to silkworms infected with *C. albicans* was able to show protection suggesting that the killing of silkworms requires active proliferation of the fungus within them. Hanaoka and colleagues [158] have also shown that mutation of the *C. albicans PTC1* gene, which previously was not reported to be associated with pathogenicity, resulted in a decreased ability of the mutant to kill silkworms. This observation suggested that novel virulence genes can be identified by using the silkworm infection model or that some genes are species-specific in terms of pathogenicity.

*C. glabrata*, is the second most common cause of candidosis and is often CO-isolated with *C. albicans*. *C. glabrata* strains are often more resistant to antifungal drugs, in particular fluconazole. *C. glabrata* also has low infectivity in the murine model and the establishment of a reproducible mouse model system is often difficult. Silkworms infected with *C. glabrata* do not die until at least 4 days post-infection. To establish an infection, a complex regime is followed, whereby the silkworm is fed a high glucose diet inducing a form of diabetes. Worms are then infected with *C. glabrata* resulting in killing within 3 days at 37 °C. Ueno and colleagues [159] generated a library of *C. glabrata* deletion strains and characterised those essential for infection using the diabetic silkworm model. This study, identified the *Cyb2* strain, a lactate dehydrogenase mutant as having decreased the ability to kill the silkworms. Interestingly, this phenotype was also replicated in the murine model [159]. The *cyb2* gene knock-out mutant in *C. glabrata* was found to have decreased ability to adapt to the intestinal tract. This study showed that *CYB2* expression was upregulated in the gut allowing *C. glabrata* to adapt to this diabetic environment allowing infection. Therefore, the silkworm model revealed a novel mechanism necessary for *C. glabrata* infection.

*C. neoformans* is a fatal fungal disease and often associated with patients with impaired immunity [151,152]. The silkworm animal model has been shown not to die after infection at 27 °C for 4 days but does die within 3 days when incubated at 37 °C. *C. neoformans* strains of serotype A are known to have higher infectivity in mammals than those of serotype D, this has been confirmed in the silkworm model [157,160]. Thus suggesting that the silkworm infection model is useful for distinguishing between *C. neoformans* strains of weak and high pathogenicity.

To date, the silkworm model has had limited exposure in *Aspergillus* research. *A. fumigatus* is an environmental filamentous fungus that causes opportunistic infections and types of pulmonary allergy. The incidence of invasive aspergillosis is increasing, is associated with rising levels of azole resistance and frequently leads to fatal infection unless treatment is started early. Silkworms injected with *A. fumigatus* at 27 °C die early but can be protected with the administration of antifungal drugs such as amphotericin B and voriconazole. In a study by Nakamura and colleagues [161], a novel antifungal agent for the treatment of *A. fumigatus* infections was identified in the silkworm model. Therefore, the silkworm model also has considerable potential to identify pathogenicity factors of *A. fumigatus* and in studies of antifungal therapy.

Other insect models with bespoke properties have also been used successfully to model infections. For example, it was shown that the virulence hierarchy expressed in mice was phenocopied when *Candida* strains were tested in *Drosophila melanogaster* [162].

### 5.3. Galleria mellonella—The Wax Moth

Other insects have also been used as convenient models for determining the virulence of fungal pathogens or assessing the efficacy of antimicrobial drugs. Often these studies give results comparable to those from using mammals. The *Galleria mellonella* larvae model has been extensively used in the study of human fungal pathogens. It has been used for many years as bait by fishermen and is consequently easy and cheap to purchase, and infection studies can generate results within 48 h. Virulence can be measured via melanisation response of the infection and subsequent death of the larvae [163,164,165]. Changes in haemocytes (immune cells) are also used as indicators of virulence and of the insects immune response to a fungal challenge [166,167]. Despite the evolutionary divergence from mammals, the *G. mellonella* immune system shares a number of structural and functional similarities with the mammalian innate immune system [166,167]. *G. mellonella* larvae have been extensively used to assess the virulence of *A. fumgiatus, C. albicans and Cryptococcus* [165,168,169]. Changes in the viability of the larvae can be easily measured relative to the virulence of the pathogens and/or lab generated mutants strains and these provide insights on how the innate immune system of mammals may respond to a fungal pathogen. Measuring changes in the density of haemocytes and the fungal burden also reveals information on the cellular immune response to the pathogen and its ability to proliferate within the host [170,171]. Assessment of the humoral immune response of larvae has also been used as a means to monitor the expression of genes for selected antimicrobial peptides [171]. The sum of these uses means that the insect can be used as a pre-screen in virulence studies that is more compatible with the 3R’s principles (Replacement, Reduction and Refinement) in biomedical ethics and can be used to establish whether a murine infection model is ultimately necessary to provide virulence-related data.

### 5.4. Murine Model—Mus musculus

The mouse remains the species of choice in virulence and immunology studies in medical mycology due to its similarity to human physiology in addition to its ease of availably and the availability of a wide range of gene delegated strains. Inbred strains of laboratory mice are the most commonly used in animal models of the clinical effects of fungal disease. These have numerous advantages over other animal models including the ability to perform repeated body fluid sampling, the availability of a variety of established drug administration methodologies, and a wide range of genetic backgrounds and the possibility of using non-invasive imaging techniques [172,173,174]. Both mice and humans have similarities in anatomy, biochemistry, pathology and genome content [175]. Both human and mouse genomes have approximately 30,000 genes encoding proteins and less than 1% of genes have no homology [175,176]. The production of genetically defined or inbred and gene knock-out strains of mice in addition to a large number of immunological and genetic tools allow infection models to mimic a wide range of human infections. Over the decades, mice models have been used as models for systemic, pulmonary and central nervous system infections for clinically relevant human fungal pathogens [177,178,179].

In general, therefore, mice represent the host species of choice for the majority of medical mycology related questions. An important difference to note however when choosing this animal model system is the differences in the native fungal microbiome, especially when studying mucosal fungal infections. For example, healthy humans are often asymptomatic when infected with *C. albicans* and other *Candida* species which can be isolated in the gastrointestinal tract, skin and genital surfaces. It has long been known that mice are not naturally colonised by *Candida* [179]. However, colonisation by *Candida* does induce similar morphological and genetic transformations as found in humans. For example, yeast cells of *C. albicans* convert spontaneously to hyphal forms in mouse tissue and the passage of *C. albicans* through the mouse GI tract induced the expression of the transcriptional regulator *WOR1*, which is associated with a phenotypic switch favouring genes involved in a commensal phenotype [180]. The mouse reproductive tract also appears not to be colonised by *Candida* species and the establishment of murine vulvovaginal candida infections requires prolonged estrogen dosing to establish a permissive environment for *Candida* growth [181]. Another important consideration in the murine model is the mouse strain background. Inbred mice can vary in susceptibility to the same fungal burden and method of administration [182,183,184]. Zaragoza and colleagues have shown that when challenged with *C. neoformans,* different inbred mice strains exhibited variation in disease initiation and progression [185]. Similarities in responses to fungal challenges have also been observed when immune-competent mice are challenged with *A. fumigatus* conidia and invasive disease does not naturally form in the lungs [186].

**Figure 3:**
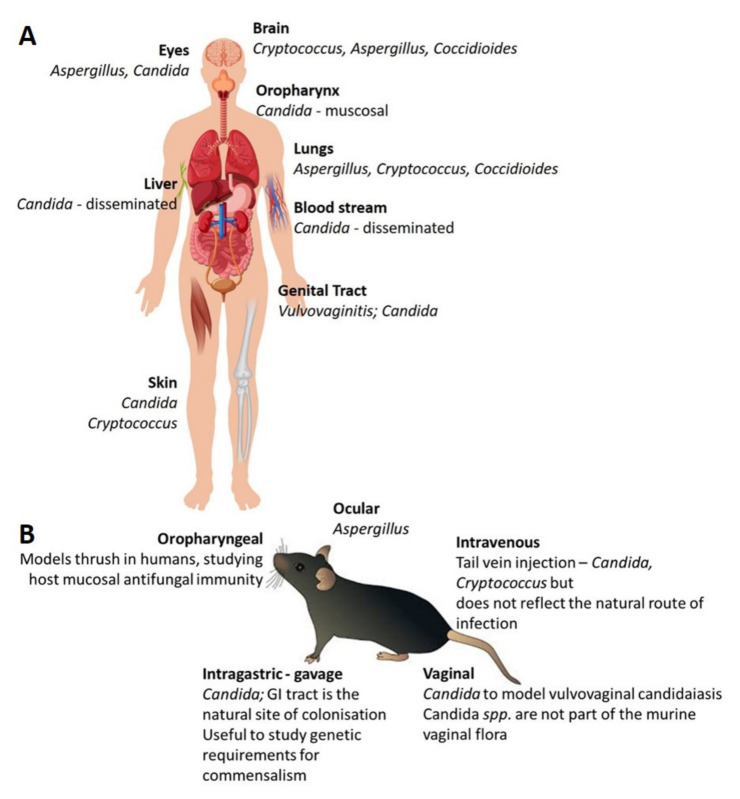
Medically relevant fungi and their normal sites of infection (**A**). The majority of fungal diseases are acquired via inhalation, skin trauma, breaches of mucosal integrity (**B**). Schematic of the common routes of fungal challenges in the murine model. (Figure adapted from [174,175,177,178], and authors own work).

The route of administration of fungal pathogens when working with murine models is also a critical variable. In general, the inoculum is administered via a physiologically relevant route of infection (Figure 3). Numerous injection and infection sites have been used with mice to model systemic, pulmonary, mucosal, gastric, superficial, dermal and central nervous system disease [178]. Systemic fungal disease can be modelled through intravenous injection of fungal cells through routes that bypass the host’s mucosal defences. It has been previously demonstrated that *C. albicans* dissemination involves mucosal damage and normally requires neutropenia [178]. The major target organ is the kidney in the mice model although other organs are also colonised. Similar to humans with systemic disease, the mice succumb to progressive sepsis and renal failure [187], due to the failure to clear fungal cells by renal macrophages and other elements of the innate immune system [188]. Fungal disease acquired via inhalation are can be recapitulated in the murine model by either intranasal or intratracheal injection of a fungal suspension. One advantage of this method of administration is that the fungal burden can be precisely quantified thus allowing for minimal experimental variability [189]. Intraperitoneal infection routes, while not recapitulating a physiological route of disease observed in humans, yield important immunological insights that are often translatable to therapeutic purposes in humans [190].

For the majority of pathogenic fungal studies, it has been clear that the host response to individual strains greatly differs in the rate of disease progression and in quality. Rizzetto et al. [190] observed in a study with *A. fumigatus* that infections caused by three different clinical strains CEA10, Af293 and Af300 resulted in varying clinical outcomes. The molecular mechanisms underlying such strain-specific differences remain unclear [191,192], but the rate of in vivo fungal growth may play a role in virulence in animal studies. Reciprocally Vaultier et al. showed that the same *Candida* strains resulted in different disease outcomes in dectin-1 −/− null mutations made in different strains of mice. Indeed, a subset of *C. albicans* strains from different clades that induce host signalling pathways via dectin-1, a C-type lectin, contributed to host protection and survival in a mouse model of systemic candidiasis [193]. In addition, *C. albicans* strains differ in pathogenesis at different sites of infection. For example, strain SC5314, isolated from a blood sample is a poor coloniser of mucosal surfaces and does not induce vulvovaginal candidiasis in female mice. This adds an additional layer of complexity to studying fungal pathogenesis as this strain is the most common background strain used to generate mutants in *C. albicans* [193].

In this section, we have set to provide an overview of the common animal models currently available to study human fungal disease. The choice of model host immune status, species, the route of infection and fungal strain being investigated are all critical variables when deciding the trajectory of the research. To draw coherent conclusions from animal work, one must consider the limitations and strengths of the animal model being used.

## 6. Conclusions

The primary aim of this review has been to provide oversight of the new variety of tools available to study human fungal pathogens. It is apparent that each methodology and infection model has unique attributes and requires careful evaluation before use and in the evaluation of the data generated. Thankfully the ability of modern molecular tools discussed above enables mutations to be made in a range of clinical backgrounds. Combinations of appropriate animal models and these new generation methodologies are, therefore, providing more sophisticated answers to be obtained from experiments designed to understand the contribution of the genotype of both the pathogen and its host to the outcome of fungal infection. With this review, we highlight just a few of the current methodologies available in the study of pathogenic fungi, many more tools are available and continuously being developed.

## Figures and Tables

**Table 1:** Tools and Resources* for studying pathogenic fungi.

Tools and Resources	*Candida albicans*	*Candida glabrata*	*Aspergillus fumigatus*	*Cryptococcus neoformans*	*Histoplasma capsulatum*	*Coccidiodes immitis*	*Blastomyces dermatitidis*	*Mucor circillenoides*
Sequenced and annotated genome	[194]	[194]	[195]	[196]	[197]	[198]	[199]	[193]
CRISPR	[195,200,201]	[202]	[16]	[18]	[203,204]	[205]	[206]	[207,208]
RNAi	[75,80]	[85]	[209]	[21,76,84]	[82]	[89]	[78]	[201]
Transposon mutagenesis	[112,123]	[210]	[116,132]	[110,136]	X	[211]	X	X
Animal Models								
Zebrafish	[140,145]	[141]	[141,142]	[143]	[212]	[212,213]	[16]	X
*Bombyx mori*—the silkworm	[157]	[155,159]	[160]	[150,156]	X	X	X	X
*Galleria mellonella*—the wax moth	[168,170,214]	[215,216]	[165,214]	[214,217]	[214,217]	X	X	[218]
*Drosophila melanogaster*	[161]	[219]	[167]	[220]	X	X	X	[90]
Murine model	[168,179,180,182,186]	[173,177,186]	[176,185]	[184]	[221]	[213]	X	[90]

*As with such a broad review topic we have chosen to discuss methods that we have utilized in the lab and are showing the most promise in our study of pathogenic fungi. This is not to be considered a full list of all tools. We have listed pathogenic fungi and tools with a reference number to further direct the reader.
